# A Reinforcement Learning Approach to Understanding Procrastination: Does Inaccurate Value Approximation Cause Irrational Postponing of a Task?

**DOI:** 10.3389/fnins.2021.660595

**Published:** 2021-09-16

**Authors:** Zheyu Feng, Asako Mitsuto Nagase, Kenji Morita

**Affiliations:** ^1^Physical and Health Education, Graduate School of Education, The University of Tokyo, Tokyo, Japan; ^2^Division of Neurology, Department of Brain and Neurosciences, Faculty of Medicine, Tottori University, Yonago, Japan; ^3^Research Fellowship for Young Scientists, Japan Society for the Promotion of Science, Tokyo, Japan; ^4^Department of Neurology, Faculty of Medicine, Shimane University, Izumo, Japan; ^5^International Research Center for Neurointelligence (WPI-IRCN), The University of Tokyo, Tokyo, Japan

**Keywords:** procrastination, value-based decision making, reinforcement learning, temporal difference learning, state representation, successor representation, dimension reduction

## Abstract

Procrastination is the voluntary but irrational postponing of a task despite being aware that the delay can lead to worse consequences. It has been extensively studied in psychological field, from contributing factors, to theoretical models. From value-based decision making and reinforcement learning (RL) perspective, procrastination has been suggested to be caused by non-optimal choice resulting from cognitive limitations. Exactly what sort of cognitive limitations are involved, however, remains elusive. In the current study, we examined if a particular type of cognitive limitation, namely, inaccurate valuation resulting from inadequate state representation, would cause procrastination. Recent work has suggested that humans may adopt a particular type of state representation called the successor representation (SR) and that humans can learn to represent states by relatively low-dimensional features. Combining these suggestions, we assumed a dimension-reduced version of SR. We modeled a series of behaviors of a “student” doing assignments during the school term, when putting off doing the assignments (i.e., procrastination) is not allowed, and during the vacation, when whether to procrastinate or not can be freely chosen. We assumed that the “student” had acquired a rigid reduced SR of each state, corresponding to each step in completing an assignment, under the policy without procrastination. The “student” learned the approximated value of each state which was computed as a linear function of features of the states in the rigid reduced SR, through temporal-difference (TD) learning. During the vacation, the “student” made decisions at each time-step whether to procrastinate based on these approximated values. Simulation results showed that the reduced SR-based RL model generated procrastination behavior, which worsened across episodes. According to the values approximated by the “student,” to procrastinate was the better choice, whereas not to procrastinate was mostly better according to the true values. Thus, the current model generated procrastination behavior caused by inaccurate value approximation, which resulted from the adoption of the reduced SR as state representation. These findings indicate that the reduced SR, or more generally, the dimension reduction in state representation, can be a potential form of cognitive limitation that leads to procrastination.

## Introduction

Delaying a task until the last minute and struggling to meet the due date is not an enjoyable thing to do. While sometimes people do this because it is inevitable or the better choice to be made, there are also other times when people voluntarily postpone the task when it could be and would better to be avoided. This irrational but voluntary delay of a course of action is known as procrastination. Previous studies have suggested that such behavior can result in not only worse academic or working performances, but also anxiety and stress in the procrastinators (e.g., Day et al., 2000; Stead et al., 2010). Procrastinators can be fully aware of the bad consequences that could potentially arise, as it was mentioned that most of procrastinators wish to reduce procrastination [mentioned in Steel (2007) by citing (O’Brien, 2002)]. The question is then raised why humans would make such seemingly irrational decisions in the first place, even when they know that such postponing could potentially worsen the situation.

Both task characteristics, such as task aversiveness and timing of rewards and punishments, and certain personality traits, such as lack of self-control and high degree of impulsivity, have been found to contribute to procrastination behavior (Steel, 2007). As it happens when the long-term and distant values give way to immediate experiences, it is also interpreted as a form of self-regulation failure (Rozental and Carlbring, 2014).

Along with these empirical findings, researchers also set out to build theoretical frameworks of procrastination. In particular, Temporal Motivation Theory (Steel and König, 2006) has been proposed as a comprehensive formulation of the mechanisms underlying procrastination. Derived from expectancy theory and hyperbolic discounting, the theory describes one’s motivation to complete a task by integrating the expectancy and the value of a task, divided by the time delay and the impulsiveness (i.e., one’s sensitivity to the delay). More recently, integrating the Temporal Motivation Theory and the self-regulation failure perspective, the temporal decision model (Zhang et al., 2019b) has been proposed. This model explicitly incorporates engagement utility or task aversiveness as an important factor related to procrastination.

Referring to these existing models, in the present study, we attempt to model procrastination from a different perspective, which is value learning and value-based decision-making. When faced with a task, whether to finish it now or to procrastinate until later is indeed a decision to be made. As mentioned above, one suggested reason for procrastination is because the procrastinators fail to prioritize values in the distant future (i.e., “delay” as in Temporal Motivation Theory), and choose immediate values instead. Task aversiveness considered in the temporal decision model, or effort cost for task engagement, should entail negative values. How humans learn and integrate these values to choose whether to procrastinate or not would thus be an interesting question in terms of value learning and value-based decision making.

Value leaning and value-based decision making, including those involving effort cost, have been widely studied in humans (e.g., Croxson et al., 2009; Kool et al., 2010; Skvortsova et al., 2014; Nagase et al., 2018; Lopez-Gamundi et al., 2021) as well as in animals (e.g., Salamone et al., 1994; Walton et al., 2003; Floresco et al., 2008; Gan et al., 2010; Cai and Padoa-Schioppa, 2019). These behaviors and their neural mechanisms have been modeled (e.g., Niv et al., 2007; Collins and Frank, 2014; Kato and Morita, 2016; Möller and Bogacz, 2019) using the framework of reinforcement learning (RL) (Sutton and Barto, 1998). It is grounded by accumulated suggestions in the past few decades that human and animal behavior can be approximated by RL models, certain neural signals appear to represent RL variables [in particular, dopamine’s encoding of reward prediction error (RPE) (Montague et al., 1996; Schultz et al., 1997) and striatal encoding of action values (Samejima et al., 2005)], and cortico-basal ganglia circuits could implement RL and action selection mechanisms (e.g., Doya, 1999; Frank et al., 2004; Lo and Wang, 2006; Khamassi and Humphries, 2012; Helie et al., 2013; Morita et al., 2016; see Niv and Montague, 2008; Lee et al., 2012 for a comprehensive review). It is thus reasonable to consider procrastination, a behavior also involving the process of value-based decision-making, on the basis of RL.

There have already been studies applying RL to procrastination (Lieder and Griffiths, 2016; Lieder et al., 2019). In their study, procrastination was considered to be a choice of the inferior option with larger proximal reward but smaller overall value due to, as suggested by the authors, cognitive limitations. They then proposed an innovative idea based on the RL theory, which was adding “pseudo-rewards” so that the optimal option will always have the maximal proximal reward (original + pseudo) and can be chosen even by the most short-sighted decision maker with cognitive limitations. The authors demonstrated in behavioral experiments with human subjects that their method successfully reduced procrastination resulting from myopic decisions.

It has, however, remained elusive exactly how (and what) cognitive limitations lead to a non-optimal choice (i.e., choice of an action whose true value is smaller than that of the optimal action). It has been suggested in the RL framework (Daw et al., 2005; Dolan and Dayan, 2013) that humans show both goal-directed and habitual behaviors, potentially approximated by model-based and model-free RL, respectively. The habitual or model-free behavior is suggested to be computationally efficient but less flexible, which in a sense reflects cognitive limitations and potentially underlies unhealthy behaviors (Story et al., 2014). Recent studies (Momennejad et al., 2017; Russek et al., 2017) have shown that humans may have also adopted an intermediate behavior between goal-directed/model-based and habitual or model-free behaviors by using a particular type of state representation named the successor representation (SR) (Dayan, 1993). As an intermediate type between model-based and model-free RL, SR-based behavior is more flexible than model-free RL, but still has some limitations as compared to fully model-based RL.

Another possible source of cognitive limitations would be dimension reduction in state representation in the brain (Gershman and Niv, 2010; Niv, 2019). As there is a tremendous number of states in the environments surrounding the humans that should not be able to be individually represented in the human brain, some sort of dimension reduction is thought to be necessary. Although low-dimensional representation can be efficient (Niv, 2019), dimension-reduced representations of states can inevitably be inadequate. For example, representing the agent’s position in the three-dimensional space by two-dimensional (*x* and *y*) coordinates cannot tell at what height (altitude) the agent exists. Inadequate state representation could cause inaccurate valuation and lead to non-optimal choice behavior.

Combining these notions, in the present study, we considered that humans may adopt a dimension-reduced version of SR (Gehring, 2015; Barreto et al., 2016; Gardner et al., 2018), in particular, the goal-based reduced SR (Shimomura et al., 2021) (see section “Methods”). We explored whether and how an RL model with the reduced SR generated procrastination behavior. More specifically, we examined if procrastinating choice, which is non-optimal in terms of true values, can nevertheless be optimal in terms of approximated values based on the approximation of state values as a linear function of features in the reduced SR in a model of Student’s behavior during vacation after a school term.

## Methods

### Modeling the Student’s Behavior in the School Term and the Vacation Period

**FIGURE 1:**
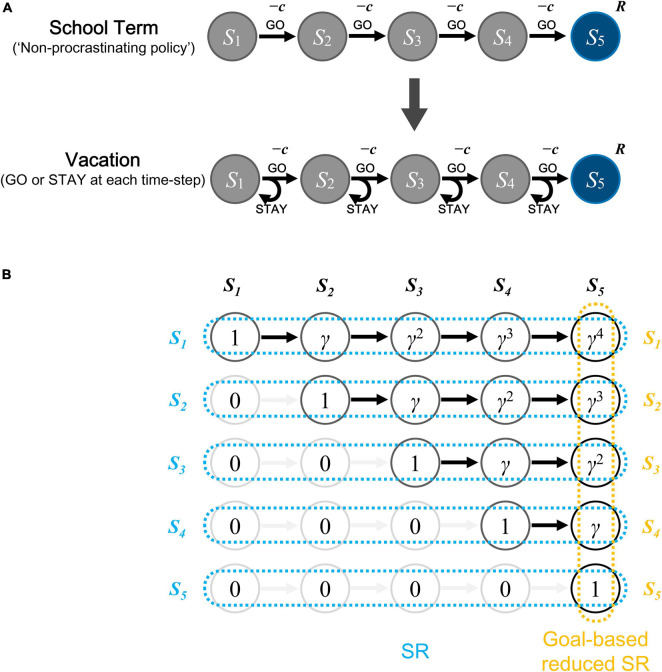
Schematic diagrams of the model and the reduced successor representation (SR). **(A)** Schematic diagrams of the model. There are 5 states for each episode, with *S*_1_ the start state and *S*_5_ the goal state. The student first experiences 20 episodes of school term, choosing “GO” at all time-steps (“non-procrastinating policy”), and then enters vacation for another 20 episodes during which the choice to “STAY” or “GO” (i.e., to procrastinate or not) is made according to the approximated values of these actions. Cost (*c*) is imposed for “GO” action and reward (*R*) is given at the goal state (*S*_5_). **(B)** Schematic diagram of the SR and the goal-based reduced SR under the policy without procrastination. The SR is the way to represent each state by a set of discounted future occupancies of all the states, i.e., to represent *S*_1_ as (1, *γ*, *γ*^2^, *γ*^3^, *γ*^4^) (where *γ* is the time discount factor), *S*_2_ as (0, 1, *γ*, *γ*^2^, *γ*^3^), *S*_3_ as (0, 0, 1, *γ*, *γ*^2^), *S*_4_ as (0, 0, 0, 1, *γ*), and *S*_5_ as (0, 0, 0, 0, 1), as indicated by the light blue marks. The goal-based reduced SR is the way to represent each state by the discounted future occupancy of only the goal state, i.e., to represent *S*_1_ as *γ*^4^, *S*_2_ as *γ*^3^, *S*_3_ as *γ*^2^, *S*_4_ as *γ*, and *S*_5_ as 1, as indicated by the orange marks.

We simulated a situation where a student experienced the school term and then started the vacation. The student, who was not allowed to procrastinate while working on assignments in the classroom during the school term, became able to choose freely whether to procrastinate while working on assignments at home during the vacation. We modeled the Student’s behavior of working on each single set of assignments (e.g., a set of math problems or short essays) by an episode of actions of an agent moving from the start state to the goal state (Figure 1A). As shown in Figure 1A, we assumed five states, and this could potentially represent the following situation, for example: each set of assignment requires about an hour of concentration (focused attention) in total, and if the student can be continuously focused for 10–15 min, s/he needs about 4–6 times of concentration, each of which could correspond to each state (except for the goal state) in our model. Notably, however, there is a study (Wilson and Korn, 2007) arguing that the frequently claimed 10–15 min duration for Student’s attention during lectures was hardly supported by the literature, and here we considered it just as an intuitive example. At each episode, the agent started from the start state, and selected at each time-step whether to go to the next state (“GO” action) with cost imposed, or stay at the current state (“STAY” action) with no cost, until reaching the goal state, where reward could be obtained (the sequential “GO” and “STAY” architecture is shared with the model of Shimomura et al. (2021) dealing with addiction, but the cost for “GO” action was introduced in the present model). The agent initially experienced 20 episodes, corresponding to the school term, under the policy of choosing “GO” at all states (i.e., without any procrastination). Subsequently, the agent experienced another 20 episodes, corresponding to the vacation period, during which the agent chose “GO” or “STAY” according to the approximated values (described below).

Notably, the “school-term/vacation” paradigm is not necessarily limited to the literal school-term or vacation. More generally, the “school-term” period could potentially simulate an “in-class” situation where the student is under supervision by the teacher or supervisor and needs to take actions under instruction. The “vacation” period, on the other hand, could potentially be analogous to a situation outside of the class where the student has the freedom to take actions.

### Goal-Based Reduced Successor Representation (SR) of States

As described in the Introduction, based on the recent suggestions of SR and dimension reduction in state representation in the brain, we assumed that the agent had acquired a dimension-reduced version of SR, specifically, the goal-based reduced SR (Shimomura et al., 2021) of each state under the policy without procrastination taken in the school term (Figure 1B). Specifically, we considered the discounted future occupancy of the final successor state (i.e., the goal state) under the policy of choosing “GO” at all states as the feature variable representing each state. Feature variable *x* for *k*-th state *S*_*k*_ (*k* = 1,…, *n*; *S*_*n*_ corresponds to the goal state, and *n* = 5 was assumed) was assumed to be:


x(Sk)=γn-k

where *γ* is the time discount factor (*γ* = 0.85 was assumed in most simulations, but we also examined a case with *γ* = 0.95). We assumed that this representation had already been established at the beginning of the initial 20 episodes of the school term that were simulated, and that it was rigid enough to remain unchanged even after the vacation period began and the agent started to also choose “STAY,” although later we also examined the case where the reduced SR was slowly updated during the vacation period.

### Approximated State Values Based on the Reduced SR, and Their Updates

The agent was assumed to approximate the state value of state *S*_*k*_ under the policy that the agent was actually taking by a linear function of the feature variable *x*:


$$\tilde{v}\left(S_{k}\right)=wx\left(S_{k}\right)$$

where $\tilde{v}\left(S_{k}\right)$ denotes the approximated state value of *S*_*k*_. Such an approximation of value function by a linear function of features has been made as a standard assumption (Montague et al., 1996; Schultz et al., 1997). It can potentially be implemented through dopamine-dependent plasticity in the brain. The coefficient *w* was updated through temporal difference (TD) learning at each time-step:


$$\delta \left(t\right)=r\left(t\right)+\gamma \tilde{v}\left(S\left(t+1\right)\right)-\tilde{v}\left(S\left(t\right)\right)$$

$$w\overset{}{\leftarrow }w+a\delta \left(t\right)x\left(S\left(t\right)\right)$$

where *δ*(*t*) denotes the TD reward prediction error (RPE), *S*(*t*) the state at time *t*, *r*(*t*) the reward or cost [modeled as negative *r*(*t*)] obtained at time *t*, and *a*, the learning rate. The reward/cost *r*(*t*) was assumed to be *R* = 1 when the agent reached the goal state, -*c* (representing the cost) when the agent chose “GO,” and 0 otherwise. The cost amount *c* was assumed to be 0.1 in most simulations, but we also examined the cases with *c* = 0, 0.01,…, 0.15. In most cases shown in the Results, the learning rate *a* was assumed to decrease over episodes (*m* = 1, …, 20):


a=0.5/(1+0.2m),

simulating habituation to the situation, in both the initial 20 episodes corresponding to the school term and the subsequent 20 episodes corresponding to the vacation period (i.e., the learning rate was assumed to once increase at the beginning of the vacation period). We also examined the cases where the learning rate was constant at 0.2 or 0.4 in both school term and vacation period. The initial value of *w* for the initial 20 episodes (the school term) was set to 0, and for the subsequent 20 episodes (the vacation period), was set to the final value of *w* at the end of the initial 20 episodes.

### Approximated Action Values Based on the Reduced SR, and Action Selection

As mentioned above, we assumed that the agent initially experienced 20 episodes during the school term under the policy of choosing “GO” at all states (i.e., without any procrastination). Subsequently, action “GO” or “STAY” was selected at each time-step according to their approximated values in a soft-max manner. We assumed that the agent computed the approximated values of the actions “GO” and “STAY” at state *S*_*k*_ (*k* = 1,., 4) by using the approximated state values under the policy that the agent was taking (described above) as follows:


$$\tilde{q}\left(S_{k},GO\right)=\gamma \tilde{v}\left(S_{k+1}\right)-c$$

$$\tilde{q}\left(S_{k},STAY\right)=\gamma \tilde{v}\left(S_{k}\right)$$

Action was then assumed to be selected according to the following probability:


$$Prob\left(A\right)=e^{b\tilde{q}\left(S_{k},A\right)}/\left\{e^{b\tilde{q}\left(S_{k},GO\right)}+e^{b\tilde{q}\left(S_{k},STAY\right)}\right\}$$

where *A* is “GO” or “STAY,” and *b* is a parameter representing the inverse of the degree of exploration (i.e., inverse temperature). In most cases shown in the Results, the inverse temperature was assumed to be constant at 20. We also examined the cases where the inverse temperature was 10 or 30.

### True State/Action Values

We explored if the agent’s behavior, determined by the approximated values based on the reduced SR, could be said to be irrational in reference to true values under the policy that the agent was taking. The true state value under the policy without procrastination for the initial 20 episodes (i.e., without “STAY”) can be exactly calculated as:


$$v\left(S_{k}\right)=\gamma^{n-k}R-C_{k}$$

where *R* represents the reward at the goal state, assumed to be 1 as mentioned above, and *C*_*k*_ stands for the summation of all the discounted future costs:


$$C_{1}=c+\gamma c+\gamma^{2}c+\gamma^{3}c$$

$$C_{2}=c+\gamma c+\gamma^{2}c$$

$$C_{3}=c+\gamma c$$

$$C_{4}=c$$

After the initial 20 episodes, the agent could freely select an action and therefore the true state values under the policy that the agent was taking should change accordingly. We considered that the agent (or the agent’s brain) could potentially estimate these values by using TD learning based on individual representation of states, in parallel with the reduced SR-based TD learning described above. Specifically, we assumed that the estimated true state value under the policy that the agent was taking $\hat{v}\left(S\right)$ was updated as:


$$\delta^{\prime }\left(t\right)=r\left(t\right)+\gamma \hat{v}\left(S\left(t+1\right)\right)-\hat{v}\left(S\left(t\right)\right)$$

$$\hat{v}\left(S\left(t\right)\right)\overset{}{\leftarrow }\hat{v}\left(S\left(t\right)\right)+a\delta^{\prime }\left(t\right)$$

with the initial values for $\hat{v}\left(S\right)$ set to the abovementioned true state values under the non-procrastinating policy. Then, given these estimated true state values, estimated true action values under the policy that the agent was taking were calculated as:


$$\hat{q}\left(S_{k},GO\right)=\gamma \hat{v}\left(S_{k+1}\right)-c$$

$$\hat{q}\left(S_{k},STAY\right)=\gamma \hat{v}\left(S_{k}\right)$$

Apart from the state/action values under the policy that the agent was taking, we can also consider the optimal state/action values, i.e., the state/action values under the optimal policy, as defined in the RL theory (Sutton and Barto, 1998). In our model with the abovementioned standard parameter values (*n* = 5, *γ* = 0.85, *R* = 1, and *c* = 0.1), the optimal policy is considered to be the non-procrastinating policy (i.e., without choosing “STAY”), because taking a “STAY” results in one more time-step discounting of the reward and all of the future costs whose (discounted) sum is positive. We considered that the agent (or the agent’s brain) could also potentially estimate the optimal action values based on individual representation of actions, for example, if Q-learning can be implemented in the brain (c.f., Roesch et al., 2007; Morita, 2014; Morita et al., 2016). On the other hand, it would be difficult for the agent to approximate the optimal action values based on the reduced SR, given that approximation of value function as a function of features is harder for off-policy, than for on-policy, learning (c.f., chapter 11 of Sutton and Barto, 2018).

### “Penalty,” or “Regret,” for Taking Action “STAY”

We also conducted separate sets of simulations, in which a “penalty” for “STAY” choice depending on the elapsed time, or an unpredictable “regret” for “STAY” choice, was added to the original model. The “penalty” term was introduced to simulate the devaluation of “STAY” choice caused by the pressure to procrastinate as the deadline approaches and/or the elapsed time increases. We added “− *p*(*t*_*v*_)*c*_*p*_” to the approximated value of “STAY” used for action selection and the true value of “STAY,” as well as the TD RPEs [*δ*(*t*) and *δ*’(*t*)] upon taking “STAY.” The parameter *c*_*p*_ controls the amount of the “penalty,” which was set to 0.1, and *p*(*t*_*v*_) is a function of time step in the vacation period (*t*_*v*_) that is 0 until *t*_*v*_ becomes a certain value, specifically, 150 time-steps, and thereafter linearly increases, specifically, according to (*t*_*v*_ − 150)/150.

The unpredictable “regret” term, on the other hand, was added to simulate “the sense of guilty” after choosing “STAY” (i.e., procrastinating). Different from the “penalty” for the “STAY” choice, the “regret” term was not added to the approximated value of “STAY” used for action selection, but only added to the true value of “STAY” as well as the TD RPEs [*δ*(*t*) and *δ*’(*t*)] upon taking “STAY,” in order to simulate that regret only showed up after “STAY” had been chosen. Specifically, we added “− *c*_*r*_” to the true value of “STAY” and the TD RPEs [*δ*(*t*) and *δ*’(*t*)] upon taking “STAY,” where *c*_*r*_ is a parameter representing the amount of the “regret,” which was set to 0.02.

### Slow Updates of the Reduced SR During Vacation

As mentioned above, so far, we assumed the goal-based reduced SR to be rigid and remaining unchanged in the vacation period. However, we also examined the case where the reduced SR was slowly updated during vacation. In the reduced SR, each state is represented by its feature variable that is the discounted future occupancy of the goal state, which can be said to be a sort of temporal proximity to the goal. As the agent changes the policy from the non-procrastinating one to the procrastinating one, the agent will need more time to reach the goal state, and thus the temporal proximity to the goal state will change (decrease). If the reduced SR changes according to the change in the policy, the feature variable for each state should also change accordingly. Such a change in the reduced SR can be done through TD learning (Shimomura et al., 2021), in the same manner as in the case of the genuine SR (Gershman et al., 2012). Specifically, the feature variable for state *S*(*t*) [i.e., *x*(*S*(*t*))] other than the goal state was updated by *α*_*SR*_*δ*_*SR*_(*t*), where *δ*_*SR*_(*t*) = *γx*[*S*(*t* + 1)] − *x*[*S*(*t*)] was the TD error for the feature variable and *α*_*SR*_ was the learning rate for this update, which was set to 0.05.

### Simulations

Simulations were conducted 10,000 times for each condition by using MATLAB.

## Results

### Learning of the Reduced SR-Based Approximated Values During the School Term

**FIGURE 2:**
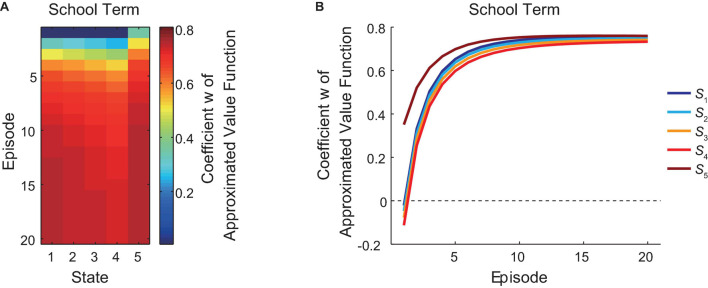
Learning of the reduced SR-based approximated values during the school term. The change of the coefficient “*w*” of the reduced SR-based approximated state value function during the school term is shown in two different ways. **(A)** The horizontal axis indicates states (*S*_1_∼*S*_5_, from the left to the right), and the vertical axis indicates the episode (1st∼20th, from the top to the bottom). The color of each square indicates the value of “*w*,” according to the right color bar, at the timing just after the agent left the corresponding state in the corresponding episode. **(B)** Each line indicates the change of “*w*” just after the agent left each state (indicated by the different colors shown on the right) over episodes (horizontal axis).

Figure 2A shows the change of the coefficient “*w*” of the reduced SR-based approximated state value function during the school term, in which the agent was assumed to take the policy of choosing “GO” all the time. The agent transitioned from state *S*_1_ (corresponding to the leftmost in Figure 2A) to the goal (state *S*_5_, rightmost) in each episode from the first episode (topmost) to the 20th episode (bottommost), and each color square in Figure 2A indicates the value of “*w*” at the timing just after the agent left the corresponding state in the corresponding episode. Figure 2B presents the same data in a different way: each line indicates the over-episode change of “*w*” just after the agent left each state. As shown in the figures, the coefficient “*w*” generally increased over the episodes, while there was a gradual decrease from *S*_1_ to *S*_4_, followed by a sharp rise at the goal state (*S*_5_), in each episode. After the update for 20 episodes, the coefficient at every state showed a tendency to gradually approach a stable value. This indicates that the agent gradually learnt, through the TD learning, the reduced SR-based approximated state values.

### Procrastination Behavior at the First Episode in the Vacation Period

**FIGURE 3:**
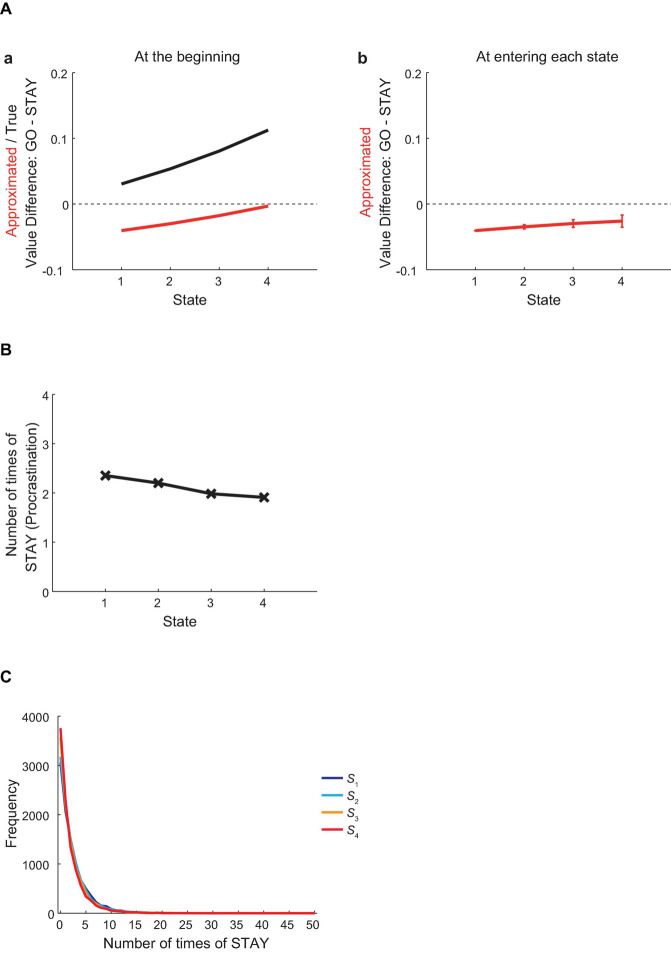
Procrastination behavior at the first episode in the vacation period. **(A)** (a) The difference between the values of actions “GO” and “STAY” at each state at the beginning of the first episode in the vacation period, for the true values under the non-procrastinating policy (black line) or the reduced SR-based approximated values (red line), averaged across simulations. (b) The difference between the approximated values of actions “GO” and “STAY” at the time when the agent initially entered each state in the first episode in the vacation period. The error bars at *S*_2_–*S*_4_ indicate the average ± standard deviation (SD) across simulations [the value at *S*_1_ indicates the value at the beginning of the first episode in the vacation period, which is the same as the one shown in (a)]. **(B,C)** The across-simulation mean **(B)** and distribution **(C)** of the number of times for the agent to choose “STAY” at each state at the first episode in the vacation period.

Figure 3Aa shows the difference between the values of actions “GO” and “STAY” at each state at the beginning of the first episode in the vacation period, for the true values under the non-procrastinating policy (i.e., choosing “GO” only) (black line) or the reduced SR-based approximated values (red line), averaged across simulations. As shown in the figure, the true value of “GO” was larger than the “STAY” value at every state, and this gap widened as the agent approached the goal state. In contrast, the reduced SR-based approximated value of “GO” was smaller than that of “STAY” at all states, though this gap narrowed as the agent approached the goal state. This contradiction indicates that the agent behaving according to the approximated values should make irrational choices of “STAY,” i.e., procrastination. Specifically, although the action “GO” had larger values than “STAY” in terms of the true values, the reduced SR-based approximated values of “GO” were smaller than those of “STAY,” and thus the agent should tend to choose “STAY” more frequently than “GO.”

Notably, the agent was assumed to update the approximated values at every time step (to approximate the values under the policy that the agent was taking) and make choices according to such continuously updated approximated values. Figure 3Ab shows the difference between the approximated values of “GO” and “STAY” at the time when the agent initially entered each state in the first episode in the vacation period, averaged across simulations. The value at *S*_1_ in this figure indicates the value at the beginning of the vacation, which is the same as the one shown in Figure 3Aa, but the average values at *S*_2_–*S*_4_ deviate from the values in Figure 3Aa, reflecting the continuous updates of the approximated values.

Figure 3B shows the mean number of times for the agent to choose “STAY” at each state at the first episode in the vacation period, averaged across simulations, and Figure 3C shows the distribution of the number of times of “STAY.” As expected from the larger approximated values of “STAY” than the values of “GO,” the agent made more than one “STAY” at every state on average. As approaching the goal state, the tendency of procrastination gradually decreased, and this can also be expected from the decrease in the difference between the approximated values of “GO” and “STAY” across states. Notably, however, as shown in Figure 3C, the distributions of the number of times of “STAY” for the four states were wide and skewed, and largely overlapped with each other.

### Changes in the Reduced SR-Based Approximated Values During the Vacation Period

**FIGURE 4:**
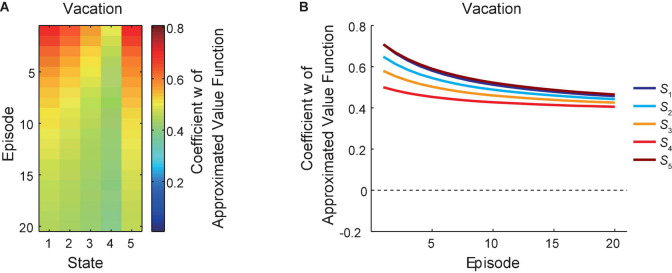
Learning of the reduced SR-based approximated values during the vacation period. The change of the coefficient “*w*” of the reduced SR-based approximated state value function during the vacation period is shown in two different ways **(A,B)**. The notations are the same as those in Figure 2.

Figure 4A shows the change of the coefficient “*w*” of the reduced SR-based approximated state value function under the policy that the agent was taking, averaged across simulations, during the vacation period, in which “GO” and “STAY” could be chosen freely. Figure 4B presents the same data in a different way: each line indicates the over-episode change of “*w*” just after the agent left each state. As shown in the figures, for each state, the coefficient “*w*” generally decreased during the vacation period, while there is again a gradual decrease from *S*_1_ to *S*_4_ and a sharp rise at the goal state (*S*_5_) in each episode. This general decrease across episodes indicates that the reduced SR-based approximated state values under the policy that the agent was taking became lowered during the vacation period, and this is considered to reflect that the policy itself gradually changed as we will see below.

### Changes in the Procrastination Behavior During the Vacation Period

**FIGURE 5:**
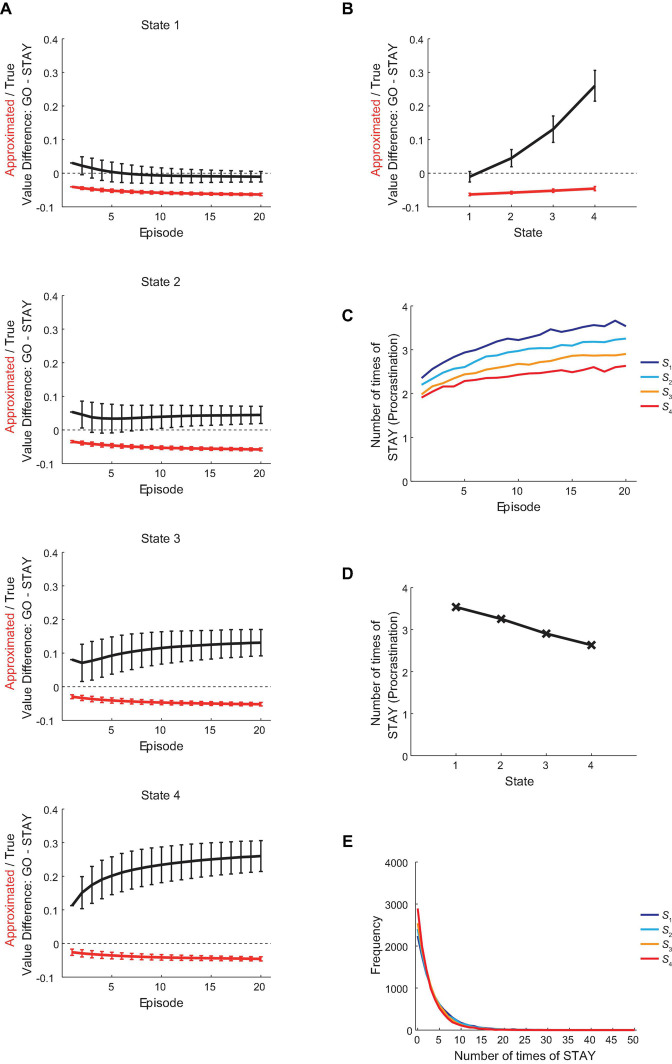
Changes in the procrastination behavior during the vacation period. **(A)** The over-episode changes of the difference between the values of actions “GO” and “STAY,” for the estimated true values under the policy that the agent was taking (black lines) and the reduced SR-based approximated values (red lines) (only the action values when the agent initially entered each state at each episode were used for calculation), except that the leftmost points of the black lines and of the red line for *S*_1_ indicate the values under the non-procrastinating policy. The error bars indicate the mean ± SD across simulations. **(B)** The difference between the values of actions “GO” and “STAY” at the time when the agent initially entered each state in the 20th episode in the vacation period, for the estimated true values (black line) or the reduced SR-based approximated values (red line). The error bars indicate the mean ± SD across simulations. **(C)** The over-episode changes of the mean number of times for the agent to choose “STAY” at each state. **(D,E)** The across-simulation mean **(D)** and distribution **(E)** of the number of times for the agent to choose “STAY” at each state at the 20th episode in the vacation period.

The red lines in Figure 5A show the over-episode changes of the difference between the reduced SR-based approximated values of actions “GO” and “STAY” under the policy that the agent was taking at entering each state, and the red line in Figure 5B shows the value difference at the 20th episode, averaged across simulations. Figure 5C shows the over-episode changes of the mean number of times for the agent to choose “STAY” at each state, and Figures 5D,E show the mean number of times to choose “STAY” at the 20th episode, averaged across simulations, and its distribution, respectively. As shown in Figure 5A, the difference between the approximated values of “GO” and “STAY” at entering every state widened over episodes (i.e., became more negative). Reflecting this, there is a clear trend of increasing in the tendency of procrastination behavior over episodes (Figure 5C). Meanwhile, the decreases in the absolute difference of the approximated values of “GO” and “STAY” and in the procrastination tendency across states within an episode remained consistent across episodes. It can thus be said that the agent’s procrastination behavior was reduced as getting closer to the goal state but was generally getting worse across the episodes.

The black lines in Figure 5A show the over-episode changes of the difference between the estimated true values of actions “GO” and “STAY” under the policy that the agent was taking at entering each state, and the black line in Figure 5B shows the value difference at the 20th episode, averaged across simulations. As shown in the bottom panel of Figure 5A, the “GO”—“STAY” difference in the estimated true values at entering *S*_4_ increased across episodes. By contrast, as shown in the top panel of Figure 5A, the “GO”—“STAY” difference at entering *S*_1_ decreased across episodes, and eventually became negative, as also appeared in Figure 5B. This indicates that at this point, choosing “STAY” at *S*_1_ has finally become a choice of a higher-(estimated)-true-value option under the procrastinating policy that the agent was actually taking. Notably, however, the optimal policy for the agent, in terms of the RL theory, is the non-procrastinating policy (choosing “GO” only) as mentioned in section “Methods,” and the true action value of “GO” under the optimal policy (i.e., the optimal action value of “GO”) was higher than that of “STAY,” as shown in the black line in Figure 3A and the leftmost point of the black line in Figure 5A, regardless of the policy that the agent was actually taking.

### Dependence of the Procrastination Behavior on the Cost of “GO” Action

**FIGURE 6:**
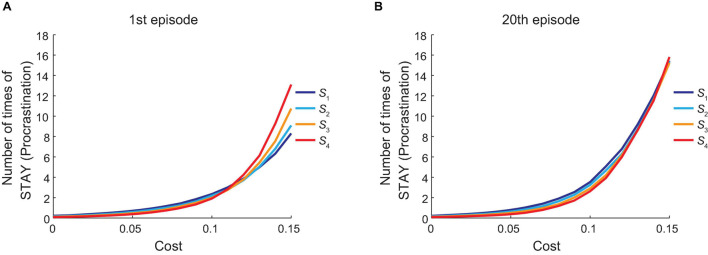
Dependence of the procrastination behavior on the cost of “GO” action. The horizontal axis indicates the amount of cost for “GO” action. The vertical axis indicates the across-simulation mean number of times for the agent to choose “STAY” at each state (indicated by the color of each line) at the first **(A)** or the 20th **(B)** episode in the vacation period.

So far, we assumed that the amount of cost imposed on each “GO” action was 0.1, which was one tenth of the amount of reward obtained at the goal. Next, we varied the amount of cost while the amount of reward was fixed and observed how the agent’s behavior changed. Figure 6A shows how the mean number of times for the agent to choose “STAY” at each state at the first episode in the vacation period, averaged across simulations, changed when the amount of cost was varied. Figure 6B shows the results for the 20th episode in the vacation period. As shown in these figures, the agent’s procrastination behavior deteriorated as cost became heavier.

### Intuitive Mechanism of Procrastination in the Model and Effects of Parameter Variations

**FIGURE 7:**
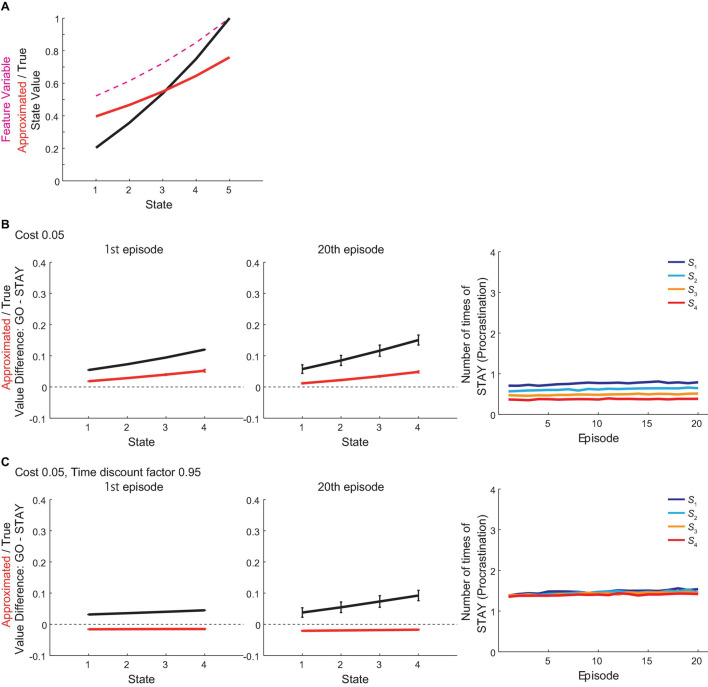
Intuitive mechanism of procrastination in the model and effects of parameter variations. **(A)** The black line indicates the true state value under the non-procrastinating policy. The red line indicates the approximated state value, which is a linear function of the feature variable of each state (magenta dashed line) based on the reduced SR, under the non-procrastinating policy. **(B)** Results of simulations where the cost was changed from the original value (0.1) to 0.05. *Left and middle panels*: The difference between the values of actions “GO” and “STAY” at the time when the agent initially entered each state in the 1st (left panel) and 20th (middle panel) episode in the vacation period, for the estimated true values (black line) or the reduced SR-based approximated values (red line). The error bars indicate the mean ± SD across simulations. *Right panel*: The over-episode changes of the mean number of times for the agent to choose “STAY” at each state. **(C)** Results of simulations where the cost *c* and the time discount factor *γ* were changed from their original values 0.1 and 0.85 to 0.05 and 0.95, respectively. Configurations are the same as those in **(B)**.

Here we explain the intuitive mechanism of how procrastination is generated in the model, and see how changes of parameters would bring to the model’s behavior by manipulating cost, time discount factor, learning rate and inverse temperature. For the true values, taking “GO” action can be said to be more advantageous than taking “STAY” action for the agent because of the following two factors: (1) if reaching the next state by taking “GO,” the reward will be less temporally discounted as the time needed to reach the goal state will decrease; and (2) if reaching the next state by taking “GO,” the remaining future costs will also decrease as the cost associated with that “GO” action will already have been paid, while “GO” is disadvantageous than “STAY” because of the associated cost. The approximated values, on the contrary, fail to incorporate the decrease in the remaining future costs properly because the approximated state value is a linear function of the feature of each state, which is discounted reward value at the goal, and is not directly related to cost amounts (although costs have indirect effects through the weight *w*). This results in that the increase in the approximated state value across states is less steep than that in the true state value (Figure 7A), and therefore the agent using the approximated values for action selection could underestimate the “GO” value, and thereby make procrastination depending on parameter values.

When the cost is small (0.05) as compared with its original amount (0.1), even for the approximated values based on the reduced SR, choosing “GO” would become more advantageous than “STAY” and would induce little procrastination behavior (Figure 7B). However, whether the cost is large or small needs to be considered relative to reward size and the rate of temporal discounting (i.e., increment of reward value from one state to next due to decrement of discounting). When the discount rate was changed to a milder level (Figure 7C, discount factor changed from the original value 0.85 to 0.95 and the cost remained 0.05 as in Figure 7B), there should be less difference in discounted reward values across states, and thereby even the small cost (0.05) made action “STAY” more advantageous than “GO” in terms of approximated values, which in turn made the agent procrastinate.

**FIGURE 8:**
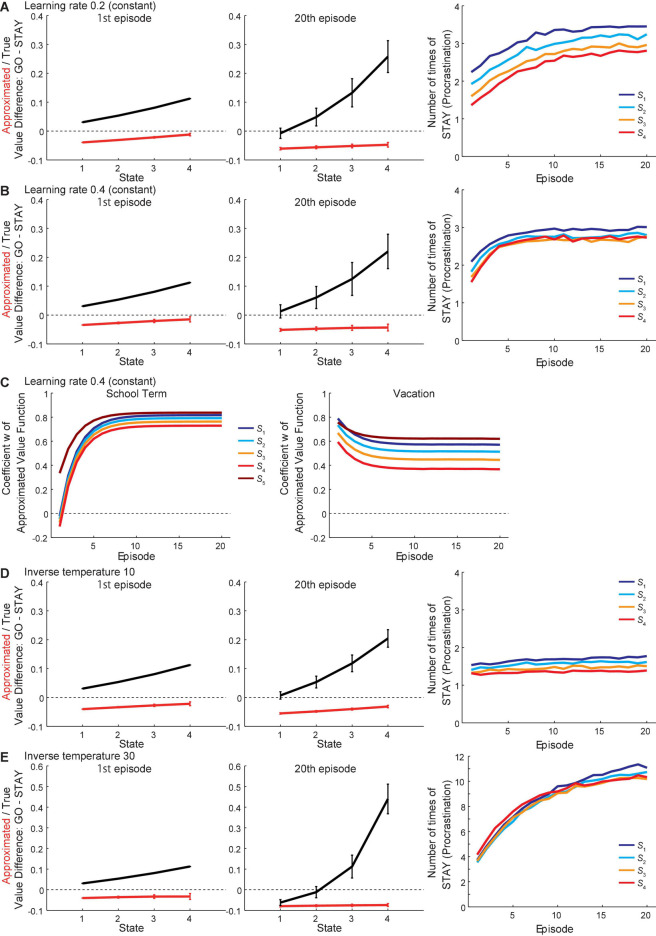
Results with different assumptions on the learning rate or the inverse temperature. **(A,B)** Results of simulations where the assumption about the learning rate *a* was changed from the original one to being constant at 0.2 **(A)** or 0.4 **(B)**. *Left and middle panels*: The difference between the values of actions “GO” and “STAY” at the time when the agent initially entered each state in the 1st (left panel) and 20th (middle panel) episode in the vacation period, for the estimated true values (black line) or the reduced SR-based approximated values (red line). The error bars indicate the mean ± SD across simulations. *Right panel*: The over-episode changes of the mean number of times for the agent to choose “STAY” at each state. **(C)** The change of the coefficient “*w*” of the reduced SR-based approximated state value function during the school term (left panel) and the vacation period (right panel) in the case where the learning rate *a* was constant at 0.4. Each line indicates the change of “*w*” just after the agent left each state over episodes. **(D,E)** Results of simulations where the inverse temperature *b* was changed from the original value (20) to 10 **(D)** or 30 **(E)**. Configurations are the same as those in **(A,B)**.

We also examined the effects of changes in the learning rate or the inverse temperature. The learning rate was originally assumed to be initially high and gradually decreasing across episodes at both school and vacation periods. When set as constant values at 0.2 or 0.4, the overall patterns of the approximated and true values were not drastically changed from the original ones (Figures 8A,B, respectively), even though the weight *w* continued to vary largely across states in the case where the learning rate was 0.4 (Figure 8C). Therefore, the assumption that learning rate decreases across episodes would not be crucial for the current model to generate procrastination behavior. Regarding the inverse temperature, when set to a smaller value (10) than the original value (20), the overall patterns of the approximated and true values were not drastically changed (Figure 8D). When set to a larger value (30) (Figure 8E), the number of times of “STAY” increased, as expected from the increased degree of exploitation, and the values in the 20th episode in the vacation look affected.

### Modifications to the Model

**FIGURE 9:**
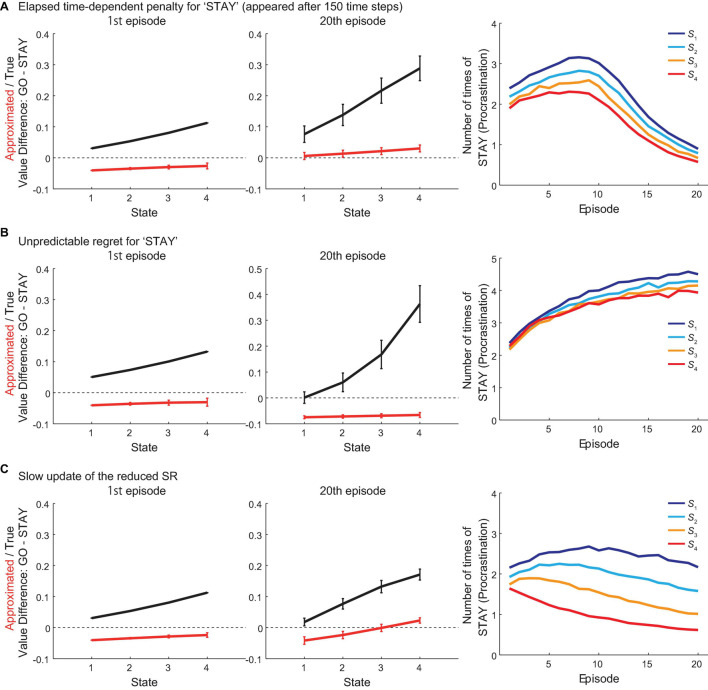
Results of simulations with modifications to the model. **(A)** Results of simulations where a “penalty” for “STAY” choice depending on the elapsed time (appeared after 150 time-steps since the beginning of the vacation period) was added to the model. *Left and middle panels*: The difference between the values of actions “GO” and “STAY” at the time when the agent initially entered each state in the 1st (left panel) and 20th (middle panel) episode in the vacation period, for the estimated true values (black line) or the reduced SR-based approximated values (red line). The error bars indicate the mean ± SD across simulations. *Right panel*: The over-episode changes of the mean number of times for the agent to choose “STAY” at each state. **(B)** Results of simulations where an unpredictable “regret” for “STAY” choice was added to the model. Configurations are the same as those in **(A)**. **(C)** Results of simulations where the reduced SR was slowly updated during the vacation period. Configurations are the same as those in **(A)**.

We also conducted separate sets of simulations, in which a “penalty” for “STAY” choice depending on the elapsed time, or an unpredictable “regret” for “STAY” choice, was added to the original model. The “penalty” was added to the approximated value of “STAY” used for action selection and the true value of “STAY,” as well as the TD RPEs upon “STAY” choice. In contrast, the unpredictable “regret” was added only to the true value of “STAY” and the TD RPEs upon “STAY” choice but not to the approximated value of “STAY” used for action selection, assuming that the agent could not foresee the regret before actually taking “STAY” and thus could not incorporate it into the approximated value of “STAY.” Figure 9A shows the results when adding the “penalty” for “STAY” choice, which appeared after 150 time-steps (since the beginning of the vacation period) and thereafter linearly increased. For all states, the number of times of “STAY” (i.e., procrastinating) initially increased, but then decreased, and the approximated values of “GO” exceeded “STAY” at the 20th episode. These results suggested that adding the “penalty” to “STAY” choice would be able to reduce procrastination. Figure 9B shows the results when adding the “regret” for “STAY” choice. The results suggested that contrary to the “penalty,” the “regret” after choosing “STAY” did not improve procrastination but even worsened the situation.

We further simulated the case where the reduced SR was slowly updated, through TD learning using the TD error of the feature variable, depending on the policy that the agent was actually taking during the vacation period. Figure 9C shows the results. Across episodes, the number of times of “STAY” at states except for *S*_4_ initially increased, but eventually became decreasing at all the states, and the approximated value of “GO” at *S*_4_ eventually exceeded the value of “STAY” at the 20th episode. These results indicated that such an update of the reduced SR could reduce procrastination.

## Discussion

This study sets out to investigate procrastination behavior from the perspective of value learning and value-based decision making. We assumed the goal-based reduced SR for state representation and modeled a series of actions and choices of a “student” during “school term” and “vacation” with cost for forward state transition and reward for reaching the goal state. The results suggested that the student, who firstly learned and updated the state value under the non-procrastinating policy during school term, soon started to procrastinate when choices can be freely made. This procrastination behavior was reduced as the student approached the goal state within the episode, but generally worsened across the episodes and with the increase of cost.

### Implications of the Present Model and the Simulation Results

Humans may make non-optimal choices due to inaccurate valuation. In the case of procrastination, procrastinators may weigh in favor of the proximal but non-optimal rewards, and against the optimal but distant reward, and this inaccurate valuation could result from cognitive limitations (Lieder and Griffiths, 2016; Lieder et al., 2019). However, exactly what sort of limitations would cause such inaccurate valuation, which further leads to procrastination, has remained elusive. In the current study, we assumed that this inaccuracy in valuation resulted from a form of state representation, which was the goal-based reduced SR. With the cost ahead and the reward in relatively distant future, the inaccurate value approximation based on the reduced SR drove the agent to procrastinate, which in turn made the reward even more distant. The estimated true value under the policy that the agent was taking, on the other hand, suggested that it was better to choose “GO” action over “STAY” action most of the times (for *S*_1_, the “STAY” value became on average slightly larger than the “GO” value as shown in Figure 5A). Although the agent first experienced episodes under the optimal policy (i.e., the non-procrastinating policy), the learned approximated values of states based on the reduced SR were already inaccurate. The inaccurate approximation of state values caused the discrepancy between the true and approximated action values and hindered the agent from making optimal decisions. Our results indicated that the reduced SR that is rigid (i.e., not easily updated) could be one of the mechanisms to explain procrastination.

As described in section “Methods,” we conducted estimation of true state values under the policy that the agent was taking during vacation through TD learning, along with TD learning of the approximated values. We considered that human brain could potentially make such an estimation of true policy-dependent state values, or even also an estimation of the true optimal action values as mentioned in section “Methods.” Possibly, such an estimation of true values could be one of the forms of value predictions in non-procrastinators. That is, it seems possible that people who can make such an estimation of true values may procrastinate less, while people who cannot might procrastinate more. Another possibility would be that human (brain) can have these different values at the same time, but the reduced SR-based approximated values can take dominance in controlling choice behavior, depending on individuals and/or conditions, or at least have some effects on choice (unless there is specific mechanism to inhibit their effects). This possibility seems to be in line with the suggestion that most procrastinators wish to reduce procrastination [mentioned in Steel (2007) by citing (O’Brien, 2002)]. It could be due to the different valuation systems in human brain yielding contradictory results, and one prevailing over the other.

Apart from the goal-based reduced-SR that we assumed, there could be other forms of state representation which can also account for cognitive limitation that leads to inaccurate valuation. In particular, state representation by low-dimensional features generally has a risk of inadequacy and thereby inaccurate valuation. Further research would be needed to test possible relations of various forms of state representation to procrastination. On the other hand, inadequate state representation and inaccurate valuation due to low-dimensional state representation can be a potential mechanism for problematic behavior, or even psychiatric disorders, other than procrastination. Recent work (Shimomura et al., 2021) proposed that rigid goal-based reduced SR can contribute to the difficulty in cessation of habitual (addictive) reward obtaining. Meanwhile, there have been reports of possible relations between behavioral addiction and procrastination (e.g., Li et al., 2020; Yang et al., 2020). Future study is desired to examine if inadequate state representation underlies the coexistence of procrastination and addiction.

### Relations to Other Studies

Previous psychological models, including the Temporal Motivation Theory (Steel and König, 2006) and the temporal decision model (Zhang et al., 2019b), have incorporated the hyperbolic type of temporal discounting in the formulation. In particular, the time inconsistency or “myopic preference reversal” (Kirby and Herrnstein, 1995), occurring in hyperbolic or quasi-exponential discounting, has been proposed to be a cause of procrastination (O’Donoghue and Rabin, 1999; Steel and König, 2006), as well as of other impulsive or unhealthy behavior (reviewed in Story et al., 2014 with a critical view). The current framework based on RL, however, showed that even only incorporating the assumed exponential discounting could generate procrastination behavior. Although it has been indicated that temporal discounting of humans and animals generally has resemblance to hyperbolic discounting (Myerson and Green, 1995; Mazur, 2001), while very hyperbolic discounting (i.e., severe discounting for a short delay) may be seen in some people and/or conditions, less hyperbolic and more exponential-like discounting could possibly be observed in others. Our model could provide a mechanistic explanation of procrastination in the latter cases.

Procrastination has been shown to be negatively correlated with scales related to self-control or planning (Steel, 2007). In our model, inaccurate value approximation caused by the reduced dimension of state features could lead to non-optimal action choices, and this could be framed as non-optimal planning. Also, it was reported (Taylor et al., 1998) that mental simulation of the process of goal reaching including detailed steps, named process simulation, facilitated performance whereas mental simulation of goal outcome, named outcome simulation, did not. Another study (Oettingen, 2012) also implicated that fantasizing or daydreaming about the desired future (i.e., the goal) could hinder the pursuit of the goal. Focusing just on the goal outcome, paying little attention to the intermediate steps, could potentially lead to a formation of, and/or reliance on, state representation based particularly on the goal state. In our model, value approximation based on the goal-based reduced SR has an inability to properly incorporate step-by-step decrement of remaining future cost, and it leads to procrastination as explained in the Results. In this regard, it is tempting to speculate that the abovementioned behavioral results for better performance with process simulation but not with outcome simulation could potentially be because the different ways of mental simulations led to different ways of state representation.

In our model, procrastination behavior was generally worsened across episodes, unless the “penalty” was added or the reduced SR was updated. In the literature, a study that objectively measured academic procrastination by examining homework initiation (Schiming, 2012) reported that generally students procrastinated more along with the progress of the academic term. However, that study examined homework during the term rather than in the vacation, and it is not sure if there are any potential links between their results and ours. Also, in our model, whereas the unpredictable “regret” coming after procrastinating did not really help with reducing procrastination, the “penalty” of procrastinating, which could potentially represent the pressure of deadline, did reduce procrastination. The latter could be regarded as an implementation of the suggested effectiveness of deadlines (Ariely and Wertenbroch, 2002), although if so, where such penalty comes from remains to be addressed.

There has not been direct evidence to support that the reduced SR is actually implemented in human brain, but there are some indirect implications. SR has been proposed to be hosted in the hippocampus and the prefrontal cortex (Russek et al., 2017; Stachenfeld et al., 2017). The possibility that the goal-based reduced SR, in addition to or instead of the genuine SR, is hosted in these regions seems in line with the observed negative correlation between the ventromedial prefrontal cortical and hippocampal blood-oxygen(oxygenation)-level-dependent (BOLD) signals and the distance to the goal (i.e., signals increase as the goal becomes closer, as in the feature variable in the goal-based reduced SR) (Balaguer et al., 2016). A resting-state functional magnetic resonance imaging (fMRI) study (Zhang et al., 2016) found positive correlation between behavioral procrastination and the regional activity of parahippocampal cortex, an area neighboring the hippocampus. Moreover, an event-related fMRI study (Zhang et al., 2019a) has shown that a decreasing coupling of hippocampus-striatum mediated the promoting effect of insufficient association between task and outcome on procrastination. These findings appear to support, to some degree, the rationale of modeling procrastination behavior under the reduced SR-based model in the present study.

### Limitations, Predictions, and Perspectives

The present study is a theoretical proposal of a hypothetical mechanism of procrastination, and its clear limitation is the absence of experiments. Further studies with human subjects will need to be undertaken to validate the model. Whether, or to what degree, humans adopt the reduced SR based on the goal state, which can be generalized to the states with immediate reward or punishment, can be tested by behavioral experiments to examine if they can adapt to changes in reward sizes more easily than to changes in reward locations (as proposed in Shimomura et al., 2021). Then, our present model predicts that the degree of adoption of the goal-based reduced SR is correlated with the degree of procrastination, especially in people whose temporal discounting is less hyperbolic (more exponential). Also, as explained in the Results, in our model, what causes procrastination (i.e., choice of action “STAY”) is that one of the benefits of taking the action “GO” (i.e., “decrement of remaining future cost”) cannot be properly taken into account if the agent resorts to approximated values based on the reduced SR. Therefore, it is expected that explicitly informing the subject of such an information (e.g., by showing remaining future cost, and its decrement by “GO” choice, by a bar indicator) would promote the “GO” choice. Our model predicts that procrastination can be mitigated by this way especially in procrastinators whose temporal discounting is not very hyperbolic.

There are also limitations of our work in terms of modeling. We assumed that the agent had acquired the reduced SR, and based on it, the approximated values were learned, but how the reduced SR itself had been acquired was not addressed. Moreover, our model assumes the school term-vacation setting, which could potentially be applied to in-class and out-of-class settings to some extents, but there should be situations that cannot be well captured by our model. Furthermore, the model does not include things that can be related to procrastination, such as alternative rewards or deadlines (although we did examine the effects of elapsed time-dependent penalty for “STAY” choice). Constructing models that can address these issues is an important future direction. Also, our model is based on the TD RL theory and the suggested representation of TD RPE by phasic dopamine signals, but it has been suggested that tonic or slowly changing dopamine signals or baseline dopamine levels may represent or relate to something different from TD RPE, in particular, action vigor or motivation (Niv et al., 2007; Howe et al., 2013; Collins and Frank, 2014; Hamid et al., 2016; Möller and Bogacz, 2019; but see also Kato and Morita, 2016; Kim et al., 2020). Also, distributional RL theory, which concerns not only the expected value but also the variance (uncertainty) or distribution of rewards, has been developed (Morimura et al., 2010; Bellemare et al., 2017; Dabney et al., 2018), and how reward uncertainty or distribution can be encoded in the basal ganglia and/or dopamine systems has been suggested (Mikhael and Bogacz, 2016; Dabney et al., 2020). It is also an interesting direction to model procrastination behavior taking these concepts beyond the conventional dopamine TD RPE hypothesis into account.

Notwithstanding the limitations, we would like to emphasize the strengths of this study. As mentioned in the Introduction, procrastination can be considered to be a form of value-based decision making, which has been extensively studied by combining behavioral, physiological, or neuroimaging experiments and RL models, leading to proposals of concrete mechanisms of how specific brain regions or neural populations encode specific variables or parameters. The present study tries to connect procrastination to the rich literature of value-based decision making, and thereby could help further our understanding of procrastination behaviors. In addition, laboratory study of procrastination can be challenging for task design, as the time for experiments is usually limited and not long enough for the participants to procrastinate. Looking from the value-based decision-making perspective, however, could potentially bring different possibilities for future practice.

## Data Availability Statement

The original contributions presented in the study are publicly available. This data can be found here: https://github.com/GigiiY/Procrastination_ReducedSR.

## Author Contributions

ZF and KM developed and elaborated the model with the reduced SR for procrastination, which KM conceived of, and conducted the simulations. Before these, AMN developed different reinforcement learning models with temporal discounting of mental effort cost for model fitting of behavior in order to explain procrastination, and discussed them with KM. ZF, AMN, and KM explored and discussed previous related studies. ZF drafted the original manuscript, and KM revised it with reference to comments of ZF and AMN. All authors contributed to the article and approved the submitted version.

## Conflict of Interest

The authors declare that the research was conducted in the absence of any commercial or financial relationships that could be construed as a potential conflict of interest.

## Publisher’s Note

All claims expressed in this article are solely those of the authors and do not necessarily represent those of their affiliated organizations, or those of the publisher, the editors and the reviewers. Any product that may be evaluated in this article, or claim that may be made by its manufacturer, is not guaranteed or endorsed by the publisher.
